# Oropharyngeal Candidiasis among Egyptian COVID-19 Patients: Clinical Characteristics, Species Identification, and Antifungal Susceptibility, with Disease Severity and Fungal Coinfection Prediction Models

**DOI:** 10.3390/diagnostics12071719

**Published:** 2022-07-15

**Authors:** Mahmoud A. F. Khalil, Mahmoud R. M. El-Ansary, Rasha H. Bassyouni, Eman E. Mahmoud, Inas A. Ali, Tarek I. Ahmed, Essam A. Hassan, Tamer M. Samir

**Affiliations:** 1 Department of Microbiology and Immunology, Faculty of Pharmacy, Fayoum University, Fayoum 63514, Egypt; maf04@fayoum.edu.eg; 2 Department of Medical Microbiology and Immunology, Faculty of Medicine, Misr University for Science and Technology (MUST), Giza 12566, Egypt; mahmoud.roshdy@must.com; 3 Department of Medical Microbiology and Immunology, Faculty of Medicine, Fayoum University, Fayoum 63514, Egypt; rhb00@fayoum.edu.eg; 4 Department of Clinical and Chemical Pathology, Faculty of Medicine, Fayoum University, Fayoum 63514, Egypt; esm02@fayoum.edu.eg; 5 Department of Family and Community Medicine, Faculty of Medicine, Misr University for Science and Technology (MUST), Giza 12566, Egypt; inas.abdelrahim@must.edu.eg; 6 Department of Internal Medicine, Faculty of Medicine, Fayoum University, Fayoum 63514, Egypt; tia00@fayoum.edu.eg; 7 Department of Tropical Medicine, Faculty of Medicine, Fayoum University, Fayoum 63514, Egypt; essam_tropical@yahoo.com; 8 Department of Microbiology and Immunology, Faculty of Pharmacy, Misr University for Science and Technology (MUST), Giza 12566, Egypt

**Keywords:** oropharyngeal candidiasis, COVID-19, SARS-Cov-2, virulence, antifungal resistance, biofilm

## Abstract

The study aimed to investigate the causative species, antifungal susceptibility, and factors associated with oropharyngeal candidiasis (OPC) among Egyptian COVID-19 patients. This is an observational, case-controlled, single-center study that included three groups: COVID-19 patients (30), COVID-19 patients with OPC (39), and healthy individuals (31). Patients’ demographic data (age, sex), laboratory tests, comorbidities, treatment, and outcomes were included. *Candida* species were isolated from COVID-OPC patient’s oropharyngeal swabs by convenient microbiological methods. Isolated strains were tested for antimicrobial susceptibility, biofilm production, aspartyl protease, and phospholipase activities. The most common respiratory symptoms reported were dyspnea (36/39; 92.4%) and cough (33/39; 84.7%). *Candida albicans* was the most common isolated species, accounting for 74.36% (29/39), followed by *Candida tropicalis* and *Candida glabrata* (15.38% and 10.26%, respectively). Amphotericin was effective against all isolates, while fluconazole was effective against 61.5%. A total of 53.8% of the isolates were biofilm producers. The phospholipase activity of *C. albicans* was detected among 58.6% (17/29) of the isolates. Significant variables from this study were used to create two equations from a regression model that can predict the severity of disease course and liability to fungal infection, with a stativity of 87% and 91%, respectively. According to our findings, COVID-19 patients with moderate to severe infection under prolonged use of broad-spectrum antibiotics and corticosteroids should be considered a high-risk group for developing OPC, and prophylactic measures are recommended to be included in the treatment protocols. In addition, due to the increased rate of fluconazole resistance, other new antifungals should be considered.

## 1. Introduction

COVID-19 is an ongoing pandemic that was epi-centered in Wuhan, Hubei Province, People’s Republic of China, and has spread to the whole world. To date, it has claimed almost 6 million deaths out of over 400 million cases [1,2,3]. Coronaviruses are large (65–125 nm in diameter) enveloped single-stranded RNA viruses that belong to the Coronaviridae family. The coronaviruses are subdivided into four subfamilies, namely alpha (α), beta (β), gamma (γ), and delta (δ) coronaviruses. The alpha lineage of coronaviruses causes asymptomatic or mild infection. In contrast, beta-linage may cause severe disease and fatalities [4]. The beta coronaviruses, namely severe acute respiratory syndrome coronavirus (SARS-CoV), H5N1 influenza-A (H1N1 2009), and Middle East respiratory syndrome coronavirus (MERS-CoV), were responsible for several worldwide pandemics. They may also cause acute lung injury (ALI) and acute respiratory distress syndrome (ARDS) [4,5,6]. COVID-19 (SARS-CoV-2) is an enveloped single-stranded RNA β-coronavirus that shares almost 80% sequence identity with SARS-CoV.

The symptoms of COVID-19 vary from mild or asymptomatic cases to severe. The most common symptoms include fever, dry cough, shortness of breath, and fatigue. Laboratory abnormalities such as lymphopenia, elevated CRP, and lactate dehydrogenase are common but not specific. Chest computed tomography scans (CT-Scan) show pneumonia, and in milder cases show ground-glass opacities, indicating less edematous fluid that improves over time. In severe cases, the density and distribution of opacities increase with signs of lobular and subsegmental consolidation [7,8]. In addition to typical clinical symptoms with other coronaviruses infections, COVID-19 tends to invade the lower airways, provoking severe immune and inflammatory responses and even a cytokine storm. The elevated levels of inflammatory mediators in a cytokine storm and lung infiltration with inflammatory cells are associated with acute lung injury (ALI) and acute respiratory distress syndrome (ARDS). The COVID-19 ALI is characterized by intra-alveolar fibrin accumulation with decreased pulmonary surfactant protein levels, leading to decreased lung compliance due to alveolar collapse. Moreover, a high-fibrin/low-surfactant intra-alveolar environment leads to lung fibrosis by fibroblast adherence and collagen deposition [7,9,10]. The patient’s prognosis is affected by the existence and severity of ALI. Early corticosteroid administration improves outcome and decreases mortality in critically ill patients suffering from cytokine storm [11,12].

Recently, Melo et al. analyzed the data of more than 9500 patients in 40 studies and pointed out several chemical and cellular biomarkers of the cytokine storm. Chemical biomarkers are interleukin-6 (IL-6), ferritin, C-Reactive Protein (CRP), procalcitonin, lactate dehydrogenase (LDH), aspartate aminotransferase (AST), creatinine, and D-dimer. While cellular markers are leukocytes, neutrophils, lymphocytes, and platelets count. Moreover, the authors considered hyperferritinemia and elevated levels of interleukin-6 as signs of systemic inflammation and consequently poor prognosis [13].

Case severity, morbidity, and mortality are affected by age, individual physiology, and pre-existing chronic conditions such as diabetes and hypertension. Moreover, microbial coinfections alter the disease pathophysiology and affect the recovery outcomes of patients [14]. Bacterial and fungal co-infections have been reported during previous influenza pandemic outbreaks. *Staphylococcus aureus*, *Streptococcus pneumonia*, *Hemophilus influenza*, *Aspergillus* spp., and *Candida* spp. were the main causative agents. Co-infections further increased morbidity and mortality rates [14,15,16,17,18]. Likewise, severely and critically ill COVID-19 patients with COVID pneumonia or developing ARDS are reported to be particularly vulnerable to bacterial and fungal superinfection, especially those hospitalized in an ICU on invasive or non-invasive ventilation [19,20]. Increased coinfection prevalence among COVID-19 patients leads to increased empirical use of broad-spectrum antibiotics as a prophylactic measure [21,22]. High prescription rates of broad-spectrum antibiotics are likely driven by clinical uncertainty and difficulty to differentiate bacterial and fungal infections from COVID-19 progression, which consequently leads to unnecessary use of antibiotics [21,22,23]. COVID-19 patients are at substantial risk of developing opportunistic fungal infections due to extensive use of antibiotics, corticosteroids, and virus-induced immune dysregulation, including lymphocytopenia [1,24,25] as well as reduced human leukocyte antigen (HLA) expression [25].

Early prediction of the disease course and liability to fungal infection will help in personalizing the treatment plan, which will improve outcomes and reduce the unnecessary use of corticosteroids and antibiotics. Recent reports have described several factors, including demographic, clinical, immunologic, hematological, biochemical, and radiographic findings, which may help clinicians predict the severity and mortality. However, the exact determinants of the COVID-19 severe disease course are not yet known. As a result, disease outcome and patient prognosis will differ from patient to patient, necessitating treatment protocol personalization [13,26].

The current study investigated the causative agent, antifungal susceptibility pattern, and factors associated with oropharyngeal candidiasis (OPC) among Egyptian COVID-19 patients. The study also developed a regression model to predict disease severity and susceptibility to fungal infection based on significant outcomes such as patient demographics, clinical, and laboratory findings.

## 2. Patients and Methods 

### 2.1. Study Design, Participants, Specimens, and Data Collection

This is an observational, case-controlled study that was conducted at the internal medicine hospital, Fayoum University Educational Hospital, from 1 April 2020 to 30 June 2021. The study included adult patients (over 18 years) admitted with moderate to critical COVID-19 and confirmed by SARS-CoV-2 RT-PCR from nasopharyngeal swabs. The classification of cases as mild, moderate, severe, and critical was performed as follows: Mild cases have mild symptoms without shortness of breath, dyspnea, or abnormal chest imaging. Moderate cases show signs and symptoms of lower respiratory infection during clinical assessment or imaging and their oxygen saturation was ≥93% in room air at sea level. Severe cases have a respiratory rate greater than 30 breaths per minute, an oxygen saturation of 93%, an arterial partial pressure of oxygen/fraction of inspired oxygen (P/F) greater than 300 mmHg, or more than 50% progression in chest radiological findings within 24 to 48 h. Critical cases have dyspnea that needs mechanical ventilation, shock, or multiple organ failure [27].

During the study period, 69 COVID-19 patients were included in the study, then divided into 2 groups as follows: those who developed OPC were included in one group (*n* = 39 patients), and the second group included 30 patients without OPC. A negative control group was included in the study and was composed of 31 healthy individuals with negative COVID PCR and no signs of OPC. Patients who developed oropharyngeal candidiasis identified by the presence of pseudomembranous structures or white plaques on the intraoral mucous layer were included in the COVID-OPC group. Oral plaques were sampled using sterile swabs that were plated on Sabouraud dextrose agar for further identification. COVID control group participants were randomly selected from COVID patients who showed no signs of OPC during hospitalization. The negative control group included healthy individuals with negative COVID PCR and no signs of OPC. The collected information included demographics (age, sex), treatment, and outcomes. Data clean-up and manual entry were performed to assure quality. Clinical and laboratory variables associated with the development of OPC and the severity of infection was determined by the comparison of the studied groups based on OPC development and disease severity. 

### 2.2. Isolation and Identification of Candida Isolates

#### 2.2.1. Isolation of *Candida* Species

Oral swabs were plated on Sabouraud dextrose agar (Oxoid, Basingstoke, UK) and incubated at 37 °C for 24–48 h. Isolated colonies were identified using standard microbiological methods (colony characters, Gram staining, urea hydrolysis, and germ tube test). White, creamy yeast-like colonies showed gram-positive budding yeast cells and pseudohyphae under light microscopic and negative urea hydrolysis test were selected for further identification [28]. Selected colonies were examined for candida species by carbohydrate assimilation in API Candida (Biomèrieux, Marcy l’Etoile, France) according to the manufacturer’s instructions.

#### 2.2.2. Phospholipase Assay

Phospholipase activities were investigated among Candida isolates as previously described [29]. Five microliters of the test isolate (10^8^ CFU/mL) were inoculated onto egg yolk agar plates and incubated at 37 °C for 48 h. The existence of a precipitation zone around the colony indicates the presence of the phospholipase enzyme. The phospholipase index (Pz) was determined as the ratio of the colony diameter to the total colony diameter and the precipitation zone. When the Pz value is one, the isolate has no phospholipase activity; when the Pz value is less than one, the isolate exhibits phospholipase activity. The lower the Pz value, the more active the phospholipase [29,30]. *C. albicans* ATCC 10231 was involved as a positive control.

#### 2.2.3. Aspartyl Protease Assay

Candida isolates’ aspartyl protease activities were evaluated as previously reported [31]. The degradation of bovine serum albumin (BSA) was measured. Ten microliters of test isolate containing 10^6^ CFU/mL were inoculated onto 1% *w*/*v* BSA (Levochem, New York, NY, USA) agar plate. The incubation conditions were maintained at 37 °C for five days. Further protease activity was suppressed by adding 20% *w*/*v* trichloroacetic acid after incubation (S D Fine-Chem Limited, Mumbai, India), and the plate was stained with 1.25% *w*/*v* amido black (Hi-Media, Mumbai, India). Proteolytic activity was demonstrated by a zone of proteolysis encircling the colony that could not be stained with amido black. The protease index (Prz) was calculated by dividing the colony diameter by the diameter of the unstained zone. A Prz value of one indicates no protease activity, while a Prz value lower than one indicates that the tested isolate expresses aspartic protease. The higher the aspartic protease activity, the lower the Prz value [32]. The assay was repeated three times for each isolate. As a positive control, *C. albicans* ATCC 10231 was used.

#### 2.2.4. Haemolysin Assay

Sabouraud dextrose agar plates enriched with 7% (*v*/*v*) blood and 3% (*w*/*v*) glucose were used to test the hemolytic activities of candida isolates [33]. The SDA medium was inoculated with (10 μL) 10^8^ cfu/mL of Candida isolates. After that, the culture plates were kept for 48 h at 37 °C in a 5% CO_2_ environment. Hemolysin production is indicated by the existence of a hemolysis zone around the colony [32]. For each isolate, the assay was repeated three times. As a control strain, *C. albicans* ATCC 90028 was involved.

#### 2.2.5. Biofilm Formation

The microtiter plate method was used to screen biofilm formation [34,35]. Sabouraud dextrose broth (SDB) (Oxoid, Basingstoke, UK) was used with a final concentration of 8% *w*/*v* glucose (Sigma-Aldrich, St. Louis, MO, USA). The candida inoculum was adjusted to approximately 3 × 10^7^ CFU/mL. Biofilm production was performed in flat-bottom microtiter plates (Hyundai Micro Co., Seoul, LTD., Korea). Sabouraud dextrose broth (180 μL) was inoculated with 20 µL cell suspension and incubated at 37 °C for 24 h. The plates were rinsed once with distilled water after incubation, and the microtiter plate was stained for 45 min with crystal violet solution. Each well was thoroughly rinsed with sterile distilled water and then destained with ethanol (95%) (Sigma-Aldrich, St. Louis, MO, USA). The destaining solution was quantified using an ELISA plate reader (Euroclone BIOTEK, Pero, Italy) at 595 nm [34,35]. Positive control for biofilm development was *C. albicans* ATCC 10231. The isolates were classified as non-biofilm producers, weak, moderate, and strong biofilm producers according to their OD590 nm values. IF OD590 nm ≤ ODc the isolate is considered (None-biofilm producers), weak biofilm producers, when OD ˂ OD590 nm ≤ 2 × ODc, moderate biofilm producers, when 2 × ODc ˂ OD590 nm ≤ 4 × ODc, and strong biofilm producers when OD590 nm ˃ 4 × ODc [34,35].

### 2.3. Antifungal Susceptibility Test

The antifungal susceptibility testing was done using the disk diffusion method in accordance with the Clinical and Laboratory Standards Institute (CLSI) M27-A3 methodology [36]. In the susceptibility tests, amphotericin B (AMB), nystatin (NYS), and fluconazole (FLU) (Oxoid, Basingstoke, UK) were utilized.

### 2.4. Statistical Analysis of the Data

Data was fed to the computer using the IBM SPSS software package version 24.0 (SPSS Inc., Chicago, IL, USA). Qualitative data were described using number and percentage. Comparison between different groups regarding categorical variables was tested using the Chi-square test. Quantitative data was described using the mean and standard deviation for normally distributed data. For normally distributed data, comparison between two independent population was done using an independent t-test, while more than two populations were analyzed using an F-test (ANOVA). The significance test results are quoted as two-tailed probabilities. The significance of the obtained results was judged at the 5% level.

## 3. Results

### 3.1. Demographics and Patient Characteristics 

During the study period, COVID-19 patients were admitted to the internal medicine department at Fayoum University hospital, out of which 39 patients developed OPC were included in the study as the COVID-OPC case group. The study included three groups: the COVID-OPC group (*n* = 39) 25 males (64.1%) and 14 females (35.9%), COVID control group (*n* = 30) 19 male (63.3%) and 11 females (36.7%), and negative control group *n* = 31, 15 males (48.4%) and 16 females (51.6%). The mean ages were (58.36 ± 9.44), (47.47 ± 14.06), and (41.16 ± 11.10) in OPC, COVID control, and negative control, respectively. Demographic data showed no significant differences in the three studied groups (*p* = 0.062) (Table 1).

### 3.2. Variables Associated with Oropharyngeal Candidiasis and Severity of Infection

**Figure 1:**
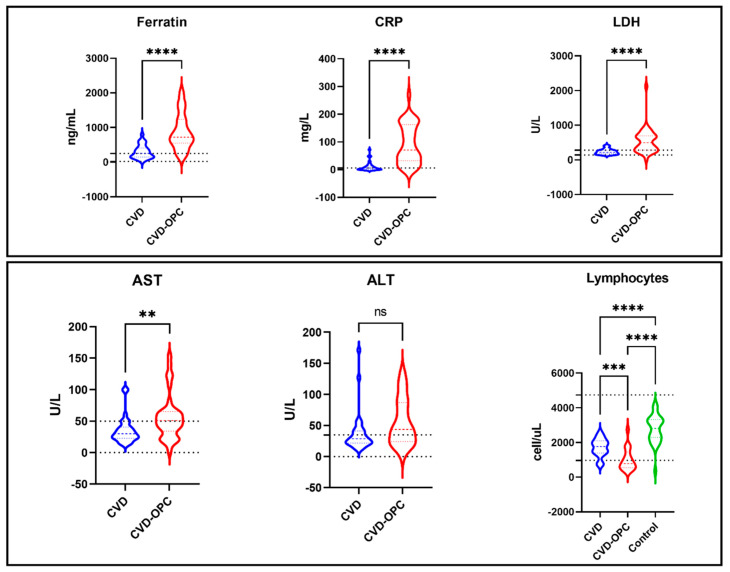
Distribution of selected laboratory tests values for the test groups. The figure illustrates the differences in some biochemical laboratory tests among the CVD group (COVID patients without oropharyngeal candidiasis), the CVD-OPC group (COVID patients with oropharyngeal candidiasis), and the control group. (CRP) C-reactive protein, (LDH) Lactate dehydrogenase, (AST) aspartate aminotransferase, (ALT) alanine transaminase. (**) indicate that *p*-value is less than 0.01 and a significant difference is present; (***) indicates that *p*-value is less than 0.001 and a significant difference is present. (****) indicates that *p*-value is less than 0.0001 and a significant difference is present. (ns) mean None significant statistical difference *p*-value is greater than 0.05.

The comparison between the three studied groups regarding demographic, laboratory, and clinical data is displayed in Table 1. Clinical data showed that the heart rate and oxygen saturation were significantly lower in the group COVID with OPC. Regarding laboratory data, most COVID-19 patients with OPC (22/39: 56.5%) showed absolute lymphocytopenia with a significantly lower absolute lymphocyte count (1015.17 ± 634.52 cell/mL) compared to (4/30; 13.3%) with a mean of (1715.33 ± 522.38 cell/mL) in the COVID-control group and (0/30) with a mean of (2777.16 ± 812.98 cell/mL) in the healthy control group (*p* = 0.001). COVID-OPC patients also showed significant hyperferritinemia (*p* = 0.001) with a mean value of (788.73 ± 469.22), that is 2 times higher than COVID patients (300.60 ± 196.05) and 5 times higher than the control group (159.16 ± 57.26). Moreover, C-reactive protein levels showed a significant 7-fold increase in the COVID-OPC group (78.16 ± 69.59) compared to the COVID group (11.68 ± 17.22) and 30 times that of the control group (2.55 ± 1.43). Likewise, LDH levels were 2 times higher in the COVID-OPC group with a mean of (539.89 ± 355.94) compared to the COVID and control groups that showed similar LDH levels (236.90 ± 78.75) and (204.32 ± 39.01), respectively. Also, the COVID-OPC group showed significantly higher levels of ALT (91.09 ± 193.26) and AST (98.41 ± 255.62) compared to other groups. Furthermore, albumin, PT, PC, INR, Urea, Ca, and D-Dimer show a significant abnormality in COVID with the OPC group more than in COVID without the OPC and control group. The distribution of selected laboratory test values for the test groups is shown in Figure 1.

Treatment protocols included the administration of corticosteroids, anticoagulants, antivirals, and antibiotics. Different classes of antibiotics were used for both studied groups, either as singles or in combinations. The commonly used antibiotics included macrolides (AZT: azithromycin), fluoroquinolones (LV: levofloxacin), cephalosporins (CFX: cefotaxime), and oxazolidinones (linezolid). Medications used for the COVID and COVID-OPC study groups are summarized in Table 2. The comparison between the severity of disease and demographics is shown in Table 3, using clinical and laboratory data. It was found that the age was higher in critical cases with high respiratory rate and low oxygen saturation. Laboratory data showed significantly elevated levels of ferratin in critical patients and severe cases compared to moderate cases (*p* = 0.001). C-reactive protein and LDH levels also showed significant increases depending on case severity, with higher levels in critical cases compared to severe and moderate cases (*p* = 0.001).

### 3.3. Prediction Models

Multivariate logistic regression analysis was used with significant variables to establish prediction models for disease severity and OPC incidence display significant prediction variables for disease severity (Table 4). These variables were used to create a model of regression to predict the severity of disease from some laboratory items. The equation (Equation (1)) from this model can facilitate the early prediction of the disease severity with 87.0% prediction accuracy. Similarly, our results display significant prediction variables for OPC incidence in COVID-19 patients (Table 5). The model can predict the incidence of OPC from common laboratory findings with 91% accuracy (Equation (2)).

Disease severity prediction Equation (1) derived from regression model of significant variables. The accuracy of the equation was 87.0%. If (a) is equal or less than one, it is likely to develop moderate COVID. If (a) is greater than one and less than two, it is likely to develop severe COVID. If (a) is greater than two, it is likely to develop critical COVID. (a) is a dependent variable (diseases Severity).

a = 4.049 − (0.033 × SPO2) − (0.005 × lymp%) + (0.006 × Mon%) + (0.0001 × CRP) + (0.001 × LDH) + (0.222 × D-dimer).(1)

OPC incidence prediction Equation (2) derived from regression model of significant variables; severity score (moderate = 1; severe = 2; critical = 3); D-dimer (negative = 0, positive = 1). If (a) is less than 0.50, no OPC is liable; and if (a) is greater than 0.50, OPC is liable. The accuracy of the equation was 91.0%.

a = 0.951 + (0.191 × severity score) + (0.025 × RR) − (0.014 × SPO2) − (0.000031 × lymph count) + (0.193 × D-dimer) + (0.00012 × LDH)(2)

### 3.4. Characterization of Candida Species

A total of 39 *candida* isolates were recovered from 39 COVID-19 patients who developed OPC. No mixed infection was detected. *Candida albicans* was the most prevalent species (29/39; 74.36%), followed by *C. tropicalis* (6/39; 15.38%), and *C. glabrata* (4/39; 10.26%). Phospholipase activity was detected among (17/29; 58.6%) of the *C. albicans* isolates. No phospholipase activity was detected among *C. glabrata* isolates, and only one isolate of *C. tropicalis* was phospholipase positive. All isolates except for (1/4; 25%) *C. glabrata* showed aspartyl protease activity to varying degrees. Regarding hemolysis, almost all the isolates showed alpha hemolysis except for a few *C. albicans* isolates (2/29; 6.9%). On the other hand, most of the isolates (21/39; 53.8%) showed positive biofilm. Most of the *C. albicans* strains were biofilm producers (17/29; 58.6%), while *C. tropicalis* and *C. glabrata* showed fewer biofilm producing isolates with (3/6; 50%) and (1/4; 25%), respectively. Biofilm production varied from weak to strong within all the isolates. The biochemical properties of isolated strains are displayed in Table 6, (Supplementary Figure S1).

### 3.5. Antifungal Susceptibility Testing

All isolates showed a high level of susceptibility to the tested antifungals. Amphotericin was the most effective antifungal with no resistance shown among the tested isolates (39/39; 100%) being sensitive to amphotericin. Nystatin was effective against all non-albicans isolates (10/10; 100%), while only (27/29; 94.8%) *C. albicans* isolates were sensitive. Fluconazole showed the least activity against all isolates; only (24/39, 61.5%) isolates were susceptible to fluconazole, while (4/39, 10.2%) isolates showed dose-dependent sensitivity, and (11/39, 28.2%) were resistant; the details are shown in Table 6.

## 4. Discussion

The COVID-19 disease course has a wide spectrum of clinical manifestations that vary from asymptomatic or mild cases to critical illness. Disease severity was attributed to several host factors [13,26,37,38]. Early prediction of disease severity course and liability to development of fungal infection will help in personalizing treatment protocols and consequently improve outcomes. Several studies have recently reported COVID-19-related fungal infections, including invasive aspergillosis and oral candidiasis [19,20,39,40]. To date, there is no clear understanding of the pathogenesis and immune response involved in COVID-19 associated fungal superinfection and candidiasis that can explain the currently rising rates. Viral-induced immune-modulation and treatment protocols with extended spectrum antibiotics and corticosteroids were proposed as potential risk factors for fungal co-infection among COVID-19 patients [19,41,42]. In this study, we investigated the patients’ variables associated with oropharyngeal candidiasis among COVID-19 patients in a case-controlled setting. We also characterized the causative agents and their antifungal susceptibility pattern. 

Immune dysregulation caused by COVID-19 has been suggested as a potential cause of increased oral fungal infection [39,41,43,44]. The result of this study showed that absolute lymphocyte count is a significant variable associated with fungal infection. The COVID OPC group showed a significantly lower absolute lymphocyte count (1015.17 ± 634.52) compared to the COVID (1715.33 ± 522.38) and control groups (2777.16 ± 812.98). Moreover, critically ill patients showed a lower relative lymphocyte count (11.60 ± 8.15) compared to moderate and severe groups (14.40 ± 9.19 and 25.13 ± 11.48), respectively. Our findings are consistent with previous studies reporting lymphocytopenia as a significant abnormality presented in the early phase of COVID-19 that plays a determinant role in prognosis and is associated with higher mortality [1,13,38]. 

In the same line, Netea et al, reported that T helper 1 and T helper 17 lymphocytes as well as innate lymphoid cells are the main players in protection against mucosal surfaces Candida infections, whereas monocytes, macrophages, and neutrophils are mainly responsible for the protection against systemic candidiasis. [45]. This agrees with the present results as normal monocyte count with an insignificant difference between the studied groups.

On the other hand, absolute neutrophils count was significantly higher in the COVID-OPC group (6267.00 ± 4177.42) compared to the COVID and control groups. Our findings of absolute lymphocytopenia with normal monocytes and neutrophils count are consistent with previous reports [1,24,25,41]. However, it is difficult to explain the liability to fungal infection with the normal or elevated levels of neutrophils and monocytes. A possible explanation for this might be the attenuated monocytes’ response toward *Candida albicans* in COVID-19 patients compared to healthy controls in ex-vivo immune stimulation assays reported by Moser et al. Moser’s observation may explain the flourishing of Oral candidiasis despite the elevated levels of monocytes. [41]. Moreover, the study by Melo et al. indicated that elevated neutrophils might indicate potential clinical deterioration and poor outcome. In our study, the critically ill patient showed higher neutrophils levels than moderate cases, which is consistent with Melo et al. findings [13]. 

The high mortality rate in critically ill COVID-19 patients has been attributed to a hyperinflammatory response and cytokine storm, which cause immune dysregulation and ARDS [7,25]. Early administration of corticosteroids in critical patients reduced 28-day mortality and improved outcomes by controlling hyper inflammation and cytokine storm [11,12]. In this study, most of the patients who developed OPC were on corticosteroids, which is consistent with previous reports showing that oral or systemic corticosteroid administration is a common cause of OPC and fungal infections [46]. 

Clinically differentiating COVID-19 infection from bacterial and fungal infection is challenging, as both share common symptoms, including cough, fever, dyspnea, and radiologic consolidation [47]. Driven by uncertainty and anxiety surrounding the epidemic, overprescribing broad-spectrum antibiotics has become widespread in the pandemic era [15,47]. Excessive use of broad-spectrum antibiotics is a major risk factor for fungal superinfection due to depletion of the normal flora that creates favorable conditions for fungi to propagate [42]. In the early stages of the pandemic, management protocols included azithromycin for its potential antiviral, anti-inflammatory, and IL-6 lowering properties [25,48]. Other antibiotics such as respiratory fluoroquinolones (moxifloxacin and levofloxacin), cephalosporins, and linezolid are sometimes also prescribed [49,50]. All patients included in this study were treated with one or more antibiotics; most were on a azithromycin/cephalosporin combination. Administration of broad-spectrum antimicrobial may contribute to OPC (Table 2). In a previous study principle, Butler et al. concluded that the routine use of azithromycin for shortening time to recovery or reducing the risk of hospitalization is unjustified. Experts from the Dutch Working Party on Antibiotic Policy concluded their study by recommending antibiotic restrictions for proven or high-likelihood COVID-19 patients who presented with radiological or inflammatory markers consistent with bacterial infection [51]. 

In the present investigation, *C. albicans* (74.36%), *C. tropicalis* (15.38%), and *C. glabrata* (10.26%) were isolated from OPC patients’ oral swabs. *Candida albicans* was the most prevalent strain in OPC patients, which is in line with previous reports [2,52]. Colonizing oral mucosa with *C. glabrata* is common in patients receiving radiation for head and neck cancers [53]. Over the past years, infection has increased by 17% and is particularly common in patients with compromised immunity or after prolonged use of broad-spectrum antibiotics [54]. The prevalence of *C. glabrata* is consistent with Salehi’s study. However, *C. tropicalis* prevalence is five times higher compared to the previous study [42]. This inconsistency can be explained by COVID associated immune dysregulation and the old age of included patients that allowed weak *C. glabrata* to establish infection without *C. albicans*. In addition, ICU hospitalization and being bedridden for a long time will enhance *C. glabrata* infection despite weak adhesion. However, due to the limited number of patients, our hypothesis is not conclusive and patient-controlled study is needed for further investigation.

In this study, isolated species showed high susceptibility levels to amphotericin and nystatin. However, fluconazole showed reduced activity towards different *candida* strains, and resistant strains were identified among the *candida* isolates. This can be attributed to the intensive use of fluconazole for the treatment of OPC. Due to immune dysregulation, including lymphocytopenia, monocyte attenuation and cytokine storm, COVID-19 is a potential risk factor for developing OPC. In addition, treatment protocols may increase the risk through extensive use of antibiotics and corticosteroids. Prediction models that can help to predict the severity of disease course and liability to fungal infections will help tailor the treatment protocols as per patient need to reduce unnecessary side effects. These models will support treatment decisions, including the early administration of corticosteroids and antifungals-based severity prediction and liability to develop fungal infection, respectively.

## 5. Conclusions

In conclusion, our study highlighted some risk factors for OPC infections among COVID-19 patients. The study has suggested that COVID-19 infection is a potential risk factor for fungal infections. Our data also revealed that COVID-19 patients with moderate to severe infection under prolonged use of broad-spectrum antibiotics and corticosteroids should be considered at high risk of developing OPC, and prophylactic measures should be included in the treatment protocols. In addition, drugs other than fluconazole should be considered due to resistance.

## Figures and Tables

**Table 1:** Comparison between the three studied groups regarding demographic, laboratory, and clinical data. (*) the *p*-value < 0.05 and a significant difference is present. HR: heart rate, (Spo2) capillary oxygen saturation, (Hb) hemoglobin, (Plt) platelet (Thrombocyte) count, (Tlc) total lymphocyte count, (ALT alanine transaminase), (AST) aspartate aminotransferase, (PT) prothrombin time, (CRP) C-reactive protein.

	COVID with OPC “*n* = 39”	COVID without OPC “*n* = 30”	Control Group “*n* = 31”	*p*-Value
Demographics				
Age	58.36 ± 9.44	47.47 ± 14.06	41.16 ± 11.10	0.062
Gender				
Male	25 (64.1%)	19 (63.3%)	15 (48.4%)	0.351
Female	14 (35.9%)	11 (36.7%)	16 (51.6%)	
Clinical signs				
Severity Mderate	4 (10.3%)	30 (100.0%)	-	0.001 *
Severe	8 (20.5%)	0 (0.0%)	-	
Critical	27 (69.2%)	0 (0.0%)	-	
SBP	116.85 ± 4.58	118.67 ± 5.71	119.00 ± 4.56	0.146
DBP	65.85 ± 3.15	63.67 ± 7.18	65.81 ± 3.09	0.116
HR	82.85 ± 4.48	85.40 ± 3.85	83.13 ± 4.57	0.040 *
Temp	38.36 ± 0.72	35.90 ± 6.79	-	0.028
RR	20.46 ± 2.39	17.10 ± 1.37	-	0.001 *
Spo2	81.56 ± 6.49	96.83 ± 0.83	-	0.001 *
Laboratory tests				
Hb	12.83 ± 2.18	12.59 ± 1.37	13.32 ± 0.75	0.187
Plt	249.11 ± 128.26	239.00 ± 83.78	277.06 ± 59.35	0.282
Tlc	8.95 ± 4.54	7.90 ± 4.03	7.79 ± 1.77	0.368
Lymp%	13.08 ± 8.46	25.41 ± 12.01	35.55 ± 7.77	0.001 *
Lymph count	1015.17 ± 634.52	1715.33 ± 522.38	2777.16 ± 812.98	0.001 *
Mono%	6.75 ± 3.52	10.16 ± 4.03	5.48 ± 2.32	0.001 *
Mono count	476.01 ± 339.45	522.67 ± 186.48	433.00 ± 221.54	0.529
ALT	91.09 ± 193.26	38.36 ± 33.07	20.55 ± 4.17	0.045
AST	98.41 ± 255.62	35.43 ± 21.01	22.94 ± 4.05	0.109
Albumin	3.01 ± 0.60	3.89 ± 0.51	4.45 ± 0.62	0.001 *
INR	1.34 ± 0.49	1.26 ± 0.46	0.95 ± 0.09	0.001 *
Urea	61.65 ± 47.43	40.50 ± 24.04	16.03 ± 2.36	0.001 *
Creatinine	1.83 ± 3.11	0.86 ± 0.39	0.54 ± 0.22	0.018
Na	132.31 ± 22.93	136.83 ± 2.26	137.03 ± 2.37	0.300
K	4.06 ± 0.87	4.09 ± 0.55	4.30 ± 0.55	0.306
Ca	9.08 ± 1.09	9.92 ± 0.75	9.36 ± 0.53	0.001 *
Ferretin	788.73 ± 469.22	300.60 ± 196.05	159.16 ± 57.26	0.001 *
CRP	78.16 ± 69.59	11.68 ± 17.22	2.55 ± 1.43	0.001 *
LDH	539.89 ± 355.94	236.90 ± 78.75	204.32 ± 39.01	0.001 *
D-Dimer				
Negative	0 (0.0%)	23 (76.7%)	31 (100.0%)	
Positive	39 (100.0%)	7 (23.3%)	0 (0.0%)	0.001 *
PCR				
Negative	0 (0.0%)	0 (0.0%)	31 (100.0%)	0.001 *
Positive	39 (100.0%)	30 (100.0%)	0 (0.0%)	

**Table 2:** Medication used for COVID and COVID-OPC patients.

Medication	COVID with OPC “*n* = 39”	COVID without OPC “*n* = 30”
Paracetamol	4 (10.26%)	9 (30%)
Vit C	37 (94.87%)	29 (96.67%)
Zinc	9 (23.08%)	29 (96.67%)
Plaqunil	9 (23.08%)	24 (80%)
Steroids	35 (89.74%)	0 (0%)
Enoxaparin	6 (15.38%)	4 (13.33%)
Oseltamivir	35 (89.74%)	19 (63.33%)
Tocilizumab	4 (10.26%)	0 (0%)
Antibiotics		
Macrolide (AZT: azithromycin)	38 (97.44%)	23 (76.67%)
Cephalosporin (CFX: Cefotaxime)	33 (84.62%)	5 (16.67%)
Oxazolidinones (LZ: Linezolid)	4 (10.26%)	0 (0%)
Fluoroquinolone (LV: levofloxacin)	3 (7.69%)	1 (3.33%)
Antibiotics combinations		
AZT-CFX	30 (76.92%)	5 (16.67%)
AZT-CFX-LZ	2 (5.13%)	0 (0%)
AZT-CFX-LV	1 (2.56%)	0 (0%)
AZT-LZ	2 (5.13%)	0 (0%)
AZT-LV	2 (5.13%)	0 (0%)

**Table 3:** Relation between different studied variables and disease severity. (*) the *p*-value < 0.05 and a significant difference is present. HR: heart rate, (Spo2) peripheral capillary oxygen saturation, (Hb) hemoglobin, (Plt) platelet (Thrombocyte) count, (Tlc) total lymphocyte count, (ALT alanine transaminase), (AST) aspartate aminotransferase, (PT) prothrombin time, (CRP) C-reactive protein.

	Moderate “*n* = 34”	Severe “*n* = 8”	Critical “*n* = 27”	*p*-Value
Age	48.97 ± 14.00	56.25 ± 12.68	58.70 ± 8.87	0.001 *
SBP	118.71 ± 5.62	117.75 ± 5.37	116.26 ± 4.23	0.183
DBP	64.24 ± 6.93	65.00 ± 3.59	65.70 ± 3.09	0.575
HR	85.03 ± 4.04	82.38 ± 3.66	83.07 ± 4.75	0.123
Temp	36.15 ± 6.41	38.45 ± 0.61	38.39 ± 0.71	0.129
RR	17.47 ± 1.81	21.38 ± 2.45	20.22 ± 2.39	0.001 *
Spo2	95.12 ± 5.03	82.50 ± 3.42	81.19 ± 7.45	0.001 *
Hb	12.66 ± 1.37	13.34 ± 2.39	12.61 ± 2.23	0.639
Plt	235.52 ± 87.74	223.57 ± 118.67	262.08 ± 132.32	0.577
Tlc	7.80 ± 3.91	6.53 ± 1.99	9.89 ± 4.93	0.083
Lymp%	25.13 ± 11.48	14.40 ± 9.19	11.60 ± 8.15	0.001 *
Lymph count	1694.48 ± 519.53	860.71 ± 390.05	1001.92 ± 687.97	0.001 *
Neutrophils	2807.40 ± 1243.65	5167.86 ± 2106.56	6783.08 ± 4665.86	0.002 *
Mono%	10.04 ± 3.95	6.73 ± 3.58	6.56 ± 3.56	0.009 *
Mono count	515.40 ± 177.90	410.00 ± 170.98	497.43 ± 388.33	0.719
ALT	39.87 ± 33.96	62.70 ± 36.59	102.25 ± 224.49	0.267
AST	36.67 ± 20.59	53.83 ± 9.30	115.04 ± 297.87	0.290
Albumin	3.88 ± 0.51	2.79 ± 0.64	2.94 ± 0.47	0.001 *
INR	1.26 ± 0.41	1.09 ± 0.13	1.40 ± 0.56	0.350
Urea	41.79 ± 24.39	41.17 ± 12.22	67.64 ± 53.56	0.036 *
Creatinine	0.89 ± 0.40	1.13 ± 0.48	2.07 ± 3.59	0.151
Na	136.41 ± 2.51	135.88 ± 3.64	131.11 ± 27.55	0.484
K	4.11 ± 0.54	4.04 ± 1.16	4.03 ± 0.84	0.913
Ca	9.93 ± 0.74	9.16 ± 0.82	8.91 ± 1.15	0.001 *
Ferretin	325.97 ± 223.46	762.50 ± 213.86	836.87 ± 530.32	0.001 *
CRP	18.17 ± 32.92	59.13 ± 64.56	85.48 ± 71.69	0.001 *
LDH	248.26 ± 90.60	390.57 ± 211.31	609.19 ± 387.57	0.001 *

**Table 4:** Multiple logistic regression analysis of different variables predicts the disease severity. The obtained equations are (a = 4.049 − (0.033 × SPO2) − (0.005 × lymp%) + (0.006 × Mon%) + (0.0001×CRP) + (0.001 × LDH) + (0.222 × D-dimer). If (a) ≤ 1 moderate, if (a) < 2 severe, and if (a) ≥ 2 critical. The accuracy of the equation was 87.0%. (a) is a dependent variable: refer to (severity). (*) mean that *p*-value is less than 0.05 and a significant difference is present.

Variables	B	Std. Error	O.R.	95.0% C.I.	t	Sig.
(Constant)	4.049	2.320			2.746	0.020 *
Age	0.002	0.009	1.00	0.65–1.25	0.275	0.785
RR	0.032	0.051	0.98	0.38–1.52	0.624	0.537
Spo2	−0.033	0.016	1.32	1.52–2.11	−2.052	0.048 *
Lymp%	−0.005	0.012	1.36	0.21–0.75	−2.436	0.046 *
Lymph count	0.000	0.000	1.001	0.65–1.65	−1.431	0.162
Neutrophils	1.537 × 10^−5^	0.000	0.91	0.62–1.88	0.514	0.611
Mono%	0.006	0.024	1.49	1.23–1.85	2.234	0.036 *
Albumin	0.010	0.231	1.003	0.65–2.11	0.042	0.966
Urea	0.0032	0.207	0.98	0.55–1.97	0.798	0.528
CRP	0.0001	0.002	1.77	1.42–2.58	−2.216	0.031 *
LDH	0.001	0.000	1.75	1.32–2.11	2.234	0.033 *
D-Dimer	0.222	0.339	2.11	1.42–1.98	2.656	0.0216 *

**Table 5:** Multiple logistic regression analysis of different variables predicting the incidence of OPC. The obtained equations are (a = 0.951 + (0.191 × severity score) + (0.025 × RR) − (0.014 × SPO2) − (0.000031 × lymph count) + (0.193 × D-dimer) + (0.00012 × LDH)). If (a) is less than 0.50, no OPC is liable; and if (a) is greater than 0.50, OPC is liable. The accuracy of the equation was 91.0%. (a) is dependent variable refer to: (OPC). (*) mean that *p*-value is less than 0.05 and a significant difference is present.

Variables	B	Std. Error	O.R.	95.0% C.I.	t	Sig.
(Constant)	0.951	0.916			3.038	0.030 *
Severity	0.191	0.080	2.04	1.01–2.91	2.393	0.020 *
Respiratory rate (RR)	0.025	0.020	2.88	0.03–0.81	2.232	0.023 *
Spo2	−0.014	0.008	2.62	1.31–3.77	−1.884	0.025 *
Lymph count	−3.188 × 10^−5^	0.000	1.81	0.12–0.82	−2.472	0.039 *
D-Dimer	0.193	0.125	2.41	1.71–4.03	2.539	0.0129 *
LDH	0.00012	0.000	2.61	1.22–3.01	−2.159	0.0451 *

**Table 6:** Characterization of candida isolates. The table shows the biochemical and antifungal sensitivity properties of the isolated candida strains. (V.STR): indicates very strong; (STR): strong and (NEG): negative; (RES): resistance; (SEN), sensitive; (SDD), sensitive dose dependent.

*Candida*	*Candida albicans* *n* = 29	*Candida tropicalis* *n* = 6	*Candida glabrata* *n* = 4
Distribution	29/39 (74.4%)	6/39 (15.4%)	4/39 (10.3%)
Hemolysis			
alpha	27 (93.1%)	6 (100%)	4 (100%)
beta	2 (6.9%)	(0%)	(0%)
Biofilm			
Non	12 (41.38%)	3 (50%)	3 (75%)
Weak	9 (31.03%)	1 (16.67%)	1 (25%)
Moderate	6 (20.69%)	1 (16.67%)	(0%)
Strong	2 (6.9%)	1 (16.67%)	(0%)
Phospholipase			
NEG	12 (41.38%)	5 (83.33%)	4 (100%)
STR	15 (51.72%)	1 (16.67%)	(0%)
V.STR	2 (6.9%)	(0%)	(0%)
Aspartyl Protease			
NEG	0 (0%)	0 (0%)	1 (25%)
STR	3 (10.34%)	3 (50%)	2 (50%)
V.STR	26 (89.66%)	3 (50%)	1 (25%)
Antifungal Sensitivity			
Fluconazole (FLU)			
SEN	18 (62.07%)	3 (50%)	3 (75%)
RES	8 (27.59%)	2 (33.33%)	1 (25%)
SDD	3 (10.34%)	1 (16.67%)	(0%)
Amphotericin B (AMB)			
SEN	29 (100%)	6 (100%)	4 (100%)
RES	0 (0%)	0 (0%)	0 (0%)
SDD	0 (0%)	0 (0%)	0 (0%)
Nystatin (NYS)			
SEN	27 (93.1%)	6 (100%)	4 (100%)
RES	(0%)	(0%)	(0%)
SDD	2 (6.9%)	(0%)	(0%)

## Data Availability

Not applicable.

## Supplementary Materials

The following supporting information can be downloaded at: https://www.mdpi.com/article/10.3390/diagnostics12071719/s1, Figure S1: Activity of different virulence factors among different Candida species. The figure illustrates the levels of biofilm, phospholipase, hemolytic, and protease activities of Candida species. (Vs): indicates very strong, (s): strong and (neg): negative.
