# Supplementary material for: Oropharyngeal Candidiasis among Egyptian COVID-19 Patients: Clinical Characteristics, Species Identification, and Antifungal Susceptibility, with Disease Severity and Fungal Coinfection Prediction Models
Source: Diagnostics (Basel). 2022 Jul 15;12(7):1719. doi: 10.3390/diagnostics12071719 (PMC9316654; doi:10.3390/diagnostics12071719)
Supplement: Supplementary file 1 [file diagnostics-12-01719-s001.zip › diagnostics-1774352-supplementary.pdf]

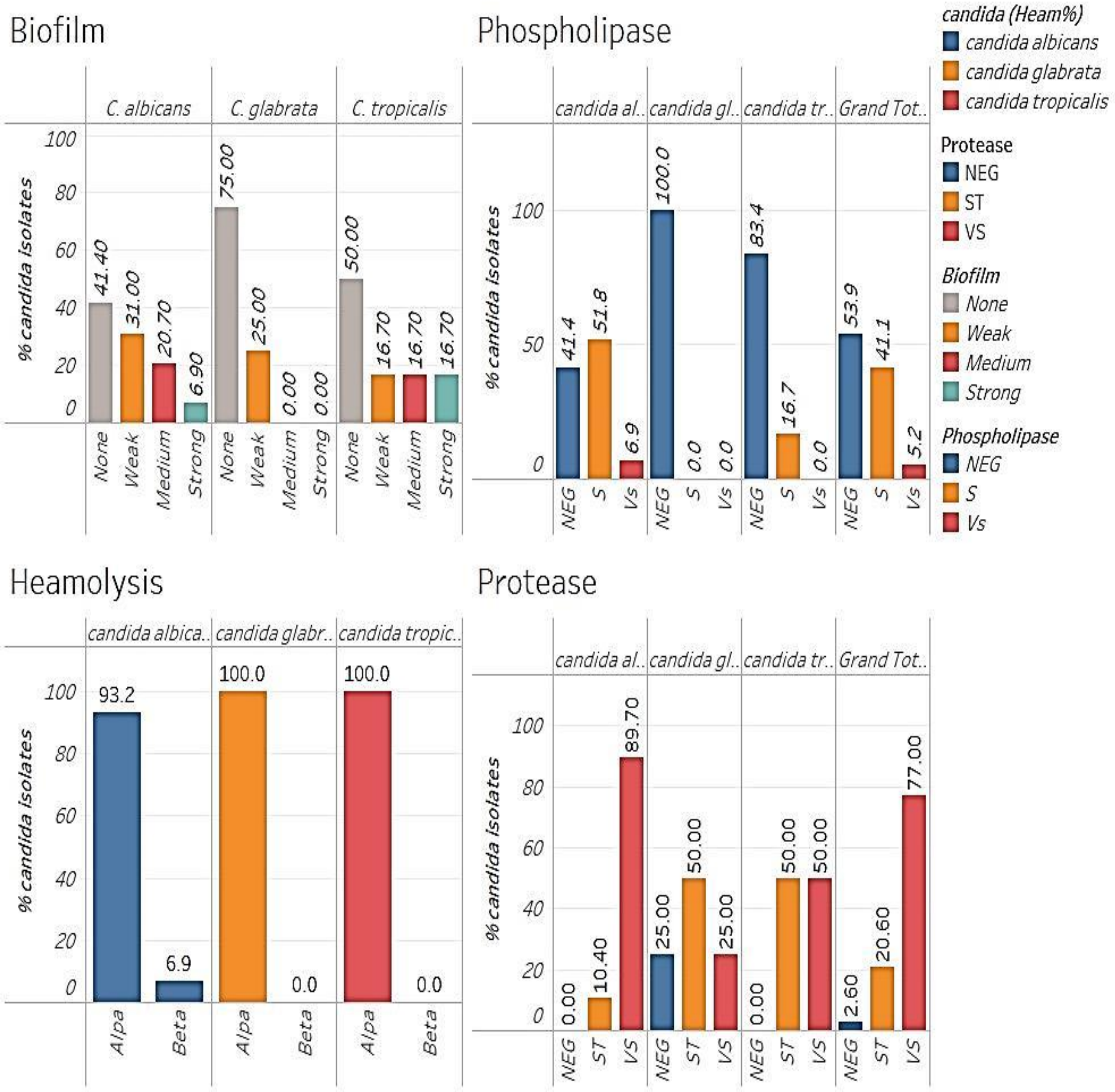

Supplementary Figure S1: Activity of different virulence factors among different *Candida* species. The figure illustrates the levels of biofilm, phospholipase, hemolytic, and protease activities of *Candida* species. (Vs): indicates very strong, (s): strong and (neg): negative.
